# The First Report of *Trichinella britovi* in Armenia

**DOI:** 10.18502/ijpa.v15i3.4212

**Published:** 2020

**Authors:** Gohar GRIGORYAN, Sargis A. AGHAYAN, Hasmik GEVORGYAN, Alexander MALKHASYAN, Isabelle VALLEE, Grégory KARADJIAN

**Affiliations:** 1. Department of Zoology, Yerevan State University, Yerevan, Armenia; 2. Molecular Parasitology Research Group, Scientific Center of Zoology and Hydroecology, The National Academy of Sciences of Armenia, Yerevan, Armenia; 3. World Wildlife Fund-Armenia, Yerevan, Armenia; 4. JRU BIPAR, Anses, Alfort Vet School, INRAE, World Animal Health Organization Collaborating Centre for Foodborne Zoonotic Parasites, Laboratory for Animal Health, Maisons-Alfort, France

**Keywords:** Lynx, Fox, *Trichinella britovi*, Armenia

## Abstract

**Background:**

More than a hundred species of mammals, birds, and reptiles are infected by nematodes of the *Trichinella* genus worldwide. Although, *Trichinella* spp. are widely distributed in neighboring countries including Georgia, Azerbaijan, Turkey and Iran, no study was conducted in Armenia since 1980’s.

**Methods:**

In 2017–2018, five muscle samples belonging to Armenian lynx, otter, wild boar, fox and wolf were tested for *Trichinella* spp. and recovered larvae were identified by multiplex PCR technique.

**Results:**

Twenty-six larvae/gram and one larva/gram were found in lynx and fox samples respectively. They were identified as *T. britovi*.

**Conclusion:**

So far only two species were identified in Armenia, *T. spiralis* and *T. pseudospiralis*, and this is the first time that *T. britovi* is reported in Armenia.

## Introduction

*Trichinella* spp. are some of the most widespread parasites infecting mammals (including Human) all over the world )1(. This zoonotic nematode includes a very broad range of host species, although only humans become clinically affected )2(. Trichinellosis is acquired by ingesting raw or under-cooked meat containing cysts (encysted L1 larvae) of *Trichinella* spp.

The first studies on the presence of *Trichinella* spp. in Armenia refer to 1950–60th when *Trichinella* spp. was detected in pigs )3(. Later in 1980–1985, wide scale investigation of *Trichinella* spp. diversity and distribution was conducted among set of wildlife and domestic animals (4). *T. spiralis* was reported in 13 different mammal species, including domestic pigs, and *T. pseudospiralis* was found in *Turdus merula* (4). *Trichinella* spp. are widely distributed in neighboring countries including Georgia, Azerbaijan, Turkey and Iran (5).

As there were no any survey conducted in Armenia after 1980’s, we decided to test five carnivorous animals muscle samples for presence of *Trichinella* spp.: lynx (*Lynx lynx dinniki*), otter (*Lutra lutra*), wild boar (*Sus scrofa*), fox (*Vulpes vulpes*) and wolf (*Canis lupus campestris*). Both of five tested samples appeared to be infected by *T. britovi* which is the first report of the species from Armenia.

## Materials and Methods

### Muscle sampling from wildlife

Collaboration with World Wildlife Fund Armenia allowed collecting some carnivorous or partly carnivorous animal muscle samples from dead animals, which included a red fox, a gray wolf, a Eurasian lynx, a Eurasian otter and a wild boar (Table 1).

**Table 1:** Muscles sampling

***ID***	***Date***	***State***	***Locality***	***Species***	***N***	***E***	***Elev***	***LPG***
GGG1	18.01.2018	Vayots Dzor	Artavan	Gray wolf (*Canis lupus*)	39°39′27″	45°37′00″	1880m	0
GGG2	26.01.2018	Vayots Dzor	Artavan	Red fox (*Vulpes vulpes*)	39°39′27″	45°37′00″	1880m	1
GGG3	05.12.2017	Hadrut	Hadrut	Wild boar (*Sus scrofa*)	39°31′00″	47°01′48″	720m	0
GGG4	10.12.2017	Vayots Dzor	Noravan k	Eurasian otter (*Lutra lutra*)	39°41’06”	45°13’59”	1550m	0
MA026	10.01.2018	Syunik	Kajaran	Eurasian lynx (*Lynx lynx*)	39°08’40”	46°08’30”	2051m	26

The sampling followed the ethical rules in force in the country and has been approved by the Yerevan State University.

Each sample size was different (25–100g), however, a minimum of 25 g was collected on carcasses. The samples were kept frozen at −20 °C until their analysis.

### Artificial digestion of muscle samples

After thawing, samples were mixed in a blender at 7000 rpm during 2 seconds. The magnetic stirrer method was then used for the artificial digestion of the mixed muscles using the European Union reference method (6). The larvae were counted in a gridded petri dish under a binocular magnifying glass.

### DNA extraction, PCR, electrophoresis

The DNA of five pooled larvae per sample was extracted using the DNA IQ System Kit (PROMEGA, DC6701) and the Tissue and Hair Extraction Kit (PROMEGA, DC6740) (7).

A multiplex PCR using the GoTaq® Hot Start Green MasterMix (PROMEGA, M5122) was performed according to published protocol (7,8). Five primer pairs were used in a multiplex PCR (7). Primer set I, ESV target locus, 5′-GTTCCATGTGAACAGCAGT-3′, 5′-CGAAAACATACGACAACTGC-3′; primer set II, ITS1 target locus, 5′-GCTACATCCTTTTGATCTGTT-3′, 5′-AGACACAATA TCAACCACAGTACA-3′; primer set III, ITS1 target locus 5′-GCGGAAGGATCATTATCGTGTA-3′, 5′-TGGATTACAAAGAAAACCATCACT-3′; primer set IV, ITS2 target locus 5′-GTGAGCGTAATAAAGGTGCAG-3′, 5′-TTCATCACACATCTTCCACTA-3′; and primer set V, ITS2 target locus 5′-CAATTGAAAACCGCTTAGCGTGTTT-3′, 5′-TGATCTGAGGTCGACATTTCC-3′.

The PCR cycles were performed as follows: a pre-denaturation and polymerase activation step at 95 °C for 2 min, then 35 amplification cycles (denaturation at 95 °C for 10 sec, hybridization at 55 °C for 30 sec and elongation at 72 °C for 30 sec), and a final elongation step at 72 °C for 5 min.

For the PCR positive controls, muscle larvae from OF-1 female mice infected by *T. spiralis*, *T. britovi*, *T. pseudospiralis* or *T. nativa* were used.

Agarose (Ozyme, LON50004) gels (2%) were prepared in TAE (2M Tris-acetate, 50 mM EDTA, pH 8.3) (Lonza, BE51216) solution with 5 ng/mL of ethidium bromide (Sigma, E1510). Electrophoresis was performed using 10 μL of PCR products with 100 pb O’Range Ruler DNA ladder (ThermoFisher Scientific, SM0653) for 30 min at 100 V.

## Results

After the artificial digestion of lynx and fox muscles, a total of 936 larvae were found in 36 g (26 LPG) and 37 larvae in 37 g (1 LPG) respectively (Table 1).

The different columns represent: the Identification (ID) of the samples, the date of sampling, the species muscles, the State, the Locality, the North (N) and East (E) coordinates, and the Elevation (Elev) where sampling occurred; in the last column, the results are given in Larvae per Gram of muscle (LPG)

**Fig. 1:**
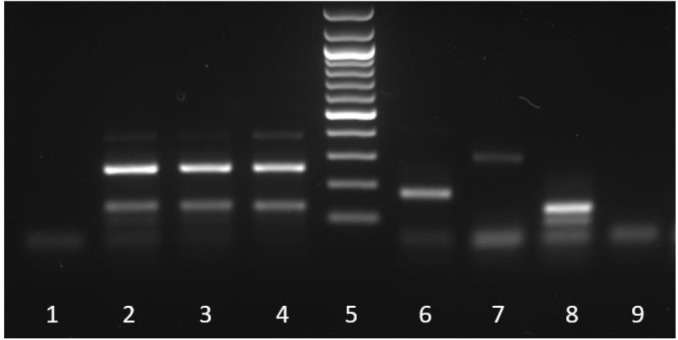
Electrophoretic profiles of multiplex PCR amplification products

The DNA analysis showed that both isolates are *Trichinella britovi* (Fig. 1). This is the first record of *T. britovi* in Armenia.

Multiplex PCR amplification products from PCR Negative Control (lane 1), DNA from *T. britovi, T.spiralis*, *T. pseudospiralis* and *T. nativa* Positive Controls (lane 2, 6, 7 and 8 respectively), from larvae found in Lynx muscles (lane3) and Red Fox Muscles (lane 4), and extraction Negative Control (lane 9). Lane 5=100 bp ladder

## Discussion

*Trichinella* is a common infection in Caucasus region including Turkey, Iran, Georgia and Azerbaijan (5). However, this is the first finding of *T. britovi* in Armenia. Previously only *T. spiralis* or *T. pseudospiralis were* reported in 1980’s (4). *T. britovi* seems to be widespread in neighboring countries.

Several outbreaks of human trichinellosis have been reported in Turkey, Antalya province (9), Izmir province and in Bursa province, all due to consumption of meat infected by *T. britovi* (10,11). Additionally, in Turkey, *T. spiralis* was found in domestic and wild boars and in pork products.

*T. britovi* was also reported in leopard (*Panthera pardus*) and wild boar (*Sus scrofa*) in Iran (12,13). Both species are known to migrate between Iran and Armenia (14). In addition, *T. britovi* was reported from number of regions south of the Caspian Sea, including neighboring to Armenia regions of Azerbaijan (15).

*Trichinella* spp. was also reported in Georgia, particularly in stone martens, jackals, red foxes and corsac foxes, and domestic cats (16) and in pigs (17). In Azerbaijan, *Trichinella* spp infections were reported in wildlife (18,19) and *T. britovi* seems to be the etiological agent in domestic and sylvatic cycles (20). Unlike Armenia, the presence of *T. britovi* was reported in all countries surrounding Armenia. As mentioned above only *T. spiralis* and *T, pseudospiralis* were detected previously (4). In case of *T. spiralis,* this could be misidentification as to that time one species in the *Trichinella* genus was known. *T. pseudospiralis* was found in one common blackbird (*Turdus Merula*) (4). This is the only species infecting birds, so it is probably the second species circulating in the country.

Our study reports for the first time the presence of *T. britovi* in lynx and fox in Armenia. Further epidemiological investigations should be conducted on carnivorous species to better understand *Trichinella* spp distribution in wild-life within the country in order to evaluate the exposure of game meat consumers in Armenia.

## Conclusion

It is the first time in Armenia *T. britovi* is reported. More research should be done to understand the current situation in Armenia, including human and wildlife.

## Ethical considerations

Ethical issues (Including plagiarism, informed consent, misconduct, data fabrication and/or falsification, double publication and/or submission, redundancy, etc.) have been completely observed by the authors.
